# The relationship between lower limb muscle volume and body mass in ambulant individuals with bilateral cerebral palsy

**DOI:** 10.1186/s12883-017-1005-0

**Published:** 2017-12-29

**Authors:** Jonathan J. Noble, Emily Chruscikowski, Nicola R. D. Fry, Andrew P. Lewis, Martin Gough, Adam P. Shortland

**Affiliations:** 1 One Small Step Gait Laboratory, Evelina London Children’s Hospital, Guy’s and St Thomas’ NHS Foundation Trust, Guy’s Hospital, London, SE1 9RT UK; 2 Division of Imaging Sciences and Biomedical Engineering, King’s College London, The Rayne Institute, 4th Floor, Lambeth Wing, St Thomas’ Hospital, London, SE1 7EH UK; 3 Department of Medical Engineering and Physics, King’s College Hospital, Denmark Hill, London, SE5 9RS UK

**Keywords:** Cerebral palsy, Muscle volume, Magnetic resonance imaging, Growth, Musculoskeletal

## Abstract

**Background:**

Individuals with cerebral palsy have smaller muscle volumes normalised to body mass than their typically developing peers. The aim of this study is to investigate the relationship between lower limb muscle volume and body mass in young people with bilateral cerebral palsy and their typically developing peers.

**Methods:**

Twenty-five participants with bilateral cerebral palsy (aged 14.7±3.0 years, GMFCS level I-III) and 25 of their typically developing peers (aged 16.8±3.3 years) took part in this study. None of the participants had undergone orthopaedic surgery, botulinum toxin injections, or serial casting in the previous year. All participants underwent magnetic resonance imaging of both lower limbs. Nine major muscles of each lower limb were individually manually segmented and the muscle volumes calculated.

**Results:**

Body mass and total lower limb muscle volume were significantly linearly related in both the cerebral palsy (R^2^ = 0.75, *p*<0.001) and typically developing (R^2^ = 0.77, *p*<0.001) groups. The slope of the relationship between muscle volume and body mass was significantly shallower in the cerebral palsy group compared to the typically developing group (*p*=0.007).

**Conclusions:**

This cross-sectional study suggests that the increase in size of lower limb muscles relative to body mass is reduced in adolescents and young adults with cerebral palsy. Longitudinal studies are required to further investigate altered muscle growth trajectories in this group and their impact on long-term mobility.

**Electronic supplementary material:**

The online version of this article (10.1186/s12883-017-1005-0) contains supplementary material, which is available to authorized users.

## Background

Cerebral palsy (CP) is the most common cause of motor disability in children, affecting approximately 2 to 3 in every 1000 live births worldwide [1–4]. CP is a group of disorders affecting the development of movement and posture, causing activity limitation, that are attributed to non-progressive disturbances that occurred in the developing foetal or infant brain [5]. The motor abilities of individuals with CP are variably affected. Hanna et al. reported that individuals with CP who could walk independently in early childhood continued to walk independently into early adulthood, but the mobility of those who relied on devices to walk tended to decline after the age of 8 years [6]. However, the scope of this study was limited to individuals up to the age of 21 years. In contrast, Day et al. classified a group of individuals at 10 years of age and a group of individuals at 25 years of age and followed them up for 15 years. In the 10 to 25 years cohort, 20% of the highest functioning group lost at least one level of mobility according to the classification of these authors, with individuals aged between 25 and 40 years of age also experiencing a decline [7]. This decline however, is even more pronounced into later life, with 75% of individuals with cerebral palsy experiencing a decline in mobility from 60 to 75 years [8]. Lower limb muscle strength relative to body mass has been shown to be a predictor of independent ambulation in children aged 8 to 19 years with CP, with muscle strength normalised to body mass decreasing over this age range [9]. Since muscle size is related to strength [10], this suggests that muscle size may be an important factor in the ambulatory status of individuals with CP.

Handsfield et al. measured the volume of 35 lower limbs muscles in 24 typically developing (TD) individuals aged 12 to 51 years and observed that the total lower limb muscle volume was significantly related to height (R^2^ = 0.64), to body mass (R^2^ = 0.85), and to height-body mass product (R^2^ = 0.92) [11]. Muscles in the lower limbs of children with CP are reduced in size relative to their body mass compared to their TD peers [12–17], with greater muscle volume deficit increasing with increasing motor impairment measured using the gross motor function classification system (GMFCS) [12]. However, little is known about muscle growth trajectories in this patient group. Research on muscle growth in CP is limited to two studies of the medial gastrocnemius. Herskind et al. performed a cross-sectional study of medial gastrocnemius muscle volume in 8 to 65 month old infants with CP and their TD peers [18]. The subjects with CP were not subcategorised into bilateral and hemiplegic subjects with the infants with CP having their more affected leg assessed [18]. This cross-sectional study suggested that medial gastrocnemius growth was reduced for infants with CP compared to their TD peers with respect to age, body mass, height, and tibial length [18]. Barber et al. recently reported a large cross sectional study of medial gastrocnemius muscle volume normalised to body mass with age in ambulant children with unilateral and bilateral CP and their TD peers aged 2 to 9 years [19]. The authors reported a reduced medial gastrocnemius muscle volume to body mass ratio in the unilateral and bilateral groups with CP compared to the TD group and that the slope of this ratio with age was reduced in the unilateral group but not in bilaterally-affected group [19]. Direct comparison of the data from these works is difficult because Herskind et al. use non-normalised volume data but Barber et al. reported a muscle volume to body mass ratio.

These studies investigated muscle growth in the medial gastrocnemius. However, muscle volume deficits vary significantly between different muscles [12, 17]. Therefore, it is possible that the estimated muscle growth reported in the cross-sectional studies by Barber et al. and Herskind et al. are not reflective of muscle growth in the lower limb musculature as a whole or over the complete period of childhood. No studies investigating muscle growth in bilateral CP have been conducted with individuals over 9 years of age. Therefore, the aim of this study is to investigate how muscle size relates to body mass in adolescence and in early adulthood with cerebral palsy compared to their typically developing peers.

In this cross-sectional study the relationship between lower limb muscle volume and body mass is investigated in a group of TD young people and in a group of young people with bilateral CP. We hypothesised that lower limb muscle volume would be linearly related to body mass in both subject groups, with the group with CP having a reduced slope between muscle volume and body mass compared to the TD group.

## Methods

### Ethics, consent and permissions

The NHS Research Ethics Service granted approval for this research (11/LO/1520,10/Y0804/83, 05/Q0704/46). All participants gave informed consent prior to data collection.

### Participants

This study was a convenience sample of individuals attending clinics at our university hospital, with consecutive patients that met the inclusion criteria invited to participate in the study. Individuals aged 10–23 years, with a diagnosis of bilateral CP, Gross Motor Function Classification System (GMFCS) levels I-III, who met the safety requirements of Magnetic Resonance Imaging (MRI) were invited to take part in this study. Patients who had undergone surgery, serial casting or botulinum toxin injections to the lower limbs within the previous year, or could not understand instructions in English were excluded from the study. TD subjects were recruited from friends and family of staff and students at our university hospital. The inclusion criteria for the TD subjects were: age 10–23 years, no neurological or musculoskeletal condition, and no previous surgery to the lower limbs. 25 individuals with bilateral CP (14.7 ± 3 years, 10.2 to 23.1 years, 19 male, GMFCS level I [*n* = 6], II [*n* = 14], and III [*n* = 5]) and 25 of their TD peers (16.8 ± 3.3 years, 19 male, 10.6 to 23.2 years) took part in this study. Muscle volume to body mass ratio data from 14 out of 25 of the CP participants and 15 out of 25 of the TD participants have been previously reported in a study comparing mean lower limb muscle volumes between individuals with CP and their TD peers [12].

### Data collection

MRI data were acquired on 1.5 T and 3.0 T Achieva systems (Philips Medical Systems, Best, The Netherlands), with a quadrature body coil. MRI images of both lower limbs of all subjects were acquired with contiguous transverse slices from above the iliac crest to below the calcaneum. All subjects lay supine on the scanner bed with their feet resting against a wooden footplate giving an approximate plantarflexion angle of 25°.

Four CP and six TD subjects were scanned using a 1.5 T MRI system using a T1 weighted turbo spin echo sequence (TE/TR = 18/1104.4 ms, number of averages = 2, echo train length = 3, 1.8 × 1.8 mm in-plane voxel size) with a quadrature body coil. Slices were collected contiguously with a slice thickness of 2 mm over the hip, knee and ankle joints and every 4 mm over the remainder of the lower limb. Image acquisition took approximately 20 min for each subject. Ten CP and nine TD subjects were also scanned using a 1.5 T system using a three point Dixon sequence (TE/TR = 4.6/13 ms, echo time shift = 1.53 ms (120 ^o^ echo phase shift), 20^o^ flip angle, 0.9 × 0.9 mm in-plane voxel size, number of averages = 2, 5 mm slice thickness) with a quadrature body coil. Eleven CP and ten TD were scanned in a 3.0 T system using a three point mDixon sequence (TE/TR = 2.11/5.2 ms, echo time shift = 0.76 ms (120 ^o^ echo phase shift), 10^o^ flip angle, 0.9 × 0.9 mm in-plane voxel size, number of averages = 2, 5 mm slice thickness) were acquired of both lower limbs.

### Image analysis

Muscle volumes were manually segmented in Osirix [20] (version 5.8.2; Pixmeo, Geneva, Switzerland) for nine muscles in both lower limbs (medial gastrocnemius, lateral gastrocnemius, soleus, tibialis anterior, semitendinosus, semimembranosus, vastus lateralis and intermedius composite, rectus femoris, and gluteus maximus). Visually, the proximal and distal endpoints of each muscle belly were identified and regions of interest were outlined on every image slice with the exception of T1 weighted scans with 2 mm slice thickness where regions were drawn on every other slice (effective slice thickness = 4 mm). The total volume for each muscle was calculated within the software as the sum of the outlined cross sectional areas multiplied by slice thickness. Muscle volumes for each muscle were averaged between legs for each individual and the muscle volume from each participant summed to produce a combined lower limb muscle volume for each participant, from here on this will be referred to as lower limb muscle volume.

To assess inter-rater and intra-rater reliability of the image analysis technique five left lower limb muscles (medial gastrocnemius, soleus, rectus femoris, and gluteus maximus) in six participants (three with CP and three TD) were analysed twice by one assessor and once by a second assessor.

### Statistical analysis

Inter-rater reliability of muscle volume segmentation technique was examined using Pearson’s correlation and an intra-class correlation coefficient (ICC). The standard error of measurement (SEM) was calculated using eq. 1, and minimal detectable change (MDC) was calculated using eq. 2. Percentage SEM and MDC were also calculated as a percentage of the average muscle volume measured by the first assessor.

1 $$ SEM={SD}^{\ast}\sqrt{1- ICC} $$

2 $$ MDC={1.96}^{\ast }{SEM}^{\ast}\sqrt{2} $$

Differences in age, body mass, height, and body mass index (BMI) between the subject groups were assessed using independent samples t-tests. Shapiro-Wilks tests of normality showed that total lower limb muscle volume and body mass can be assumed to be normally distributed (*p* > 0.05). Pearson’s correlations were performed to assess the linear relationship of limb muscle volume with body mass in the TD and CP groups. Analysis of co-variance (ANCOVA) was employed to investigate whether lower limb muscle volume was significantly related to subject group, age, or body mass. The ANCOVA had subject group and sex as fixed factors and age and body mass as covariates with the model including the interaction between subject group and body mass to test whether there was a significant difference in the slopes of the relationship of total lower limb muscle volume and body mass between the groups. The ANCOVA was repeated for each of the nine muscles under study. Significance was set at *p* = 0.05 for all statistical tests. All statistical tests were performed using SPSS (Version 20.0; IBM SPSS, Chicago, USA).

## Results

The physical characteristics of the participants are summarised in Table 1. The group with CP was significantly younger (*p* = 0.028), shorter (*p* = 0.001) and lighter (*p* = 0.001) than the TD group. The differences in mean BMI between the groups was close to significant (*p* = 0.055), with the group with CP having a lower BMI. Some of the participants in the group with CP had undergone previous surgical or pharmaceutical intervention to their lower limbs as part of their clinical management: thirteen participants had undergone muscular and/or bony orthopaedic surgery; seven participants had undergone previous orthopaedic surgery and botulinum toxin injections; and five participants had not undergone any previous interventions. The group median number of orthopaedic intervention was 1, range 0 to 3, and the group median number of botulinum toxin injections was 0, range 0 to 3. Of the individuals who had undergone previous surgery, the median number of interventions was 1 (range 1 to 3) and of the individuals who had undergone previous botulinum toxin injections the median number of botulinum toxin interventions was 2 (range 1 to 3). Details of the intervention history for each participant are given in the Additional file 1.

**Table 1:** Participant physical characteristics

	Group with CP	TD Group
Number of subjects	25	25
Age (years)	14.7 ± 3.0*	16.8 ± 3.3*
Age range (years)	10.2–23.1	10.6–23.2
Gender (m,f)	19, 6	19, 6
Body mass (kg)	51.1 ± 16.3*	65.1 ± 12.5*
Height (m)	1.59 ± 0.14*	1.72 ± 0.13*
BMI	20.0 ± 4.0	21.7 ± 2.1
Ethnicity	24 white caucasion, 1 mixed.	24 white caucasion, 1 black.
GMFCS level I	6	–
GMFCS level II	14	–
GMFCS level III	5	–

Mean ± standard deviation for the physical characteristics of the CP and TD groups and number of CP participants in each GMFCS level. * Denotes significant difference between groups (*p* < 0.05)

**Fig. 1:**
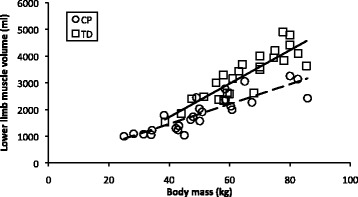
Lower limb muscle volume against body mass for the group with CP (circle, dashed line, R^2^ = 0.75, muscle volume = 37.2*body mass − 26.9) and TD group (square, sold line R^2^ = 0.77, muscle volume = 62.57*body mass − 786.6)

Lower limb muscle volume was significantly linearly related to body mass in both the TD (R^2^ = 0.77, *p* < 0.001) and CP groups (R^2^ = 0.75, *p* < 0.001). Figure 1 shows lower limb muscle volume against body mass for both participant groups. Muscle volume was significantly dependent on body mass (p = <0.001). Subject group (*p* = 0.238), sex (*p* = 0.108) and age (*p* = 0.406) were not significant factors. The slope of the linear regression equation for the group with CP was 40.6% shallower than for the TD group (see Table 2). We found that there was a significant difference between the slopes of the muscle volume – body mass relationship in the two subject groups (*p* = 0.007).

**Table 2:** Linear regression coefficients and gradients

Muscle	Subject group	Linear regression of muscle volume vs. body mass		CP Percentage difference in gradient to TD group
		R^2^	Gradient	
Combined	TD	0.77**	62.57	40.6*
	CP	0.75**	37.18	
Gluteus Maximus	TD	0.72**	15.82	38.7*
	CP	0.80**	9.69	
Rectus Femoris	TD	0.56**	4.64	45.9^†^
	CP	0.56**	2.51	
Vastus lateralis and intermedius composite	TD	0.69**	20.2	35.0^†^
	CP	0.75**	13.12	
Semi-membranosus	TD	0.64**	3.4	39.1
	CP	0.50**	2.07	
Semi-tendinosis	TD	0.44**	3.33	57.4^†^
	CP	0.40**	1.42	
Tiblias Anterior	TD	0.61**	2.05	55.6*
	CP	0.70**	0.91	
Medial Gastrocnemius	TD	0.59**	3.44	45.9*
	CP	0.49**	1.86	
Lateral Gastrocnemius	TD	0.76**	2.52	79.8*
	CP	0.48**	0.51	
Soleus	TD	0.72**	7.15	36.1^†^
	CP	0.53**	4.57	

Linear regression coefficients and gradients of the best-fit line for muscle volume against body mass for all of the muscles investigated together with the percentage difference in gradient of the CP group relative to the TD group. ** denotes *p*-value <0.001. *denotes *p*-value <0.05. ^†^denotes *p* < 0.1

**Fig. 2:**
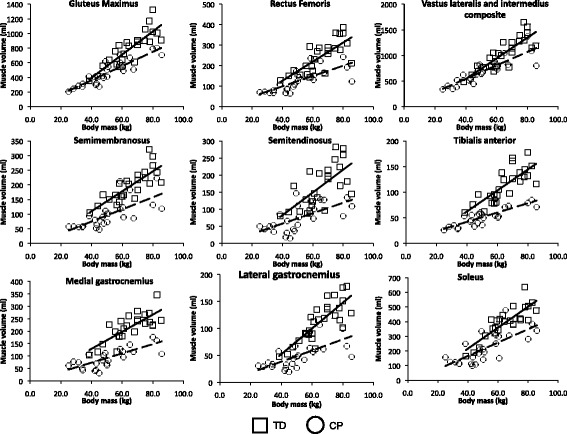
Muscle volume against body mass in the TD group (square, solid line) and group with CP (circle, dashed line) for all muscle investigated. The linear regression coefficient and gradients of the lines of best-fit are given in Table 2

Figure 2 depicts muscle volume against body mass for the individual muscles analysed in this study. Table 2 summarises the linear regression coefficients and gradients of the linear regression equations for muscle volume against body mass for all of the muscles investigated together with the percentage difference in gradient of the group with CP relative to the TD group. There was a significant linear relationship between muscle volume and body mass for all muscles in both subject groups (R^2^ = 0.4 to 0.8, *p* < 0.001). The slope of the linear regression equation was smaller in the group with CP compared to the TD group for all muscles investigated, with an average percentage reduction of 47%, ranging from 36 to 80% deficit compared to the TD group. Performing an ANOVA found that the difference in slope of the linear regression equations for muscle volume against body mass between the subject groups was statistically significant in four of the muscles investigated (gluteus maximus (*p* = 0.14); tibialis anterior (*p* = 0.003); lateral gastrocnemius (*p* < 0.001); and medial gastrocnemius (*p* = 0.034)), and not significant in 5 of the muscles investigated. However, of these 5 statistically insignificant results, four were close to significance (rectus femoris (*p* = 0.053); vastus lateralis and intermedius composite (*p* = 0.051); semitendinosus (*p* = 0.058); and soleus (*p* = 0.064).

The results of the image analysis inter-rater repeatability are given in Table 3. Pearson’s correlation of the measurements performed by assessor one and two were significant for all muscles (p < 0.001) and ranged from 0.968 for the medial gastrocnemius to 0.999 for gluteus maximus. The ICC of inter-rater repeatability were greater than 0.984 for all muscles. Percentage SEM ranged from 1.46 for rectus femoris to 4.49% for medial gastrocnmius and percentage MDC ranged from 4.04% to 12.44% for the muscles investigated.

**Table 3:** Results of the repeatability analysis of the image analysis technique for muscle volume measurement

	GMax	RF	ST	Sol	MG	Combined
Average volume (ml)	506.82	167.94	109.24	232.59	127.64	1144.23
Standard deviation (ml)	159.31	54.69	65.53	77.66	45.27	360.10
Correlation coefficient	0.999*	0.996*	0.993*	0.995*	0.968*	0.993*
ICC	0.997*	0.998*	0.995*	0.997*	0.984*	0.997*
SEM (ml)	8.73	2.45	4.63	4.25	5.73	19.72
Percentage SEM (%)	1.72	1.46	4.24	1.83	4.49	1.72
MDC (ml)	24.19	6.78	12.84	11.79	15.87	54.67
Percentage MDC (%)	4.77	4.04	11.76	5.07	12.44	4.78

Average volume and standard deviation of muscle volume from assessor 1. Percentage SEM and MDC calculated as the percentage of the average muscle volume measured by assessor 1. *GMax* gluteus maximus, *RF* rectus femoris, *ST* semi-tendinosis, *Sol* soleus, *MG* medial gastrocnemius

*Denotes *p* < 0.001

## Discussion

In groups of TD young people and young people with bilateral CP, body mass explained much of the variation in muscle volume both for a computed total muscle volume and for most individual muscles. The slope of the relationship between muscle volume and body mass was significantly shallower in the group with CP both for total muscle volume and for many of the individual muscles. The slopes of the relationships between muscle volume and body mass varied between lower limb muscles. This variability for muscles in the lower limb is in agreement with previous studies reporting heterogeneity of muscle size deficits in individuals with CP [12, 17].

The relative contributions of body mass and age to muscle size are difficult to quantify due to the co-variance of these candidate explanatory variables. In our analysis of variance, subject age did not explain, independently of body mass, variation in muscle volume. This was also true if we performed the analysis on the subgroup of participants aged below 17 years (see Additional file 1). This is an interesting result as it is a common belief that muscle volume increases with age in childhood until skeletal maturity. Our data suggests body mass, not age, is the principal explanatory factor of muscle volume during development between 10 and 16 years of age.

Our results are somewhat in contrast to those of Barber et al., who, in a large cross-sectional study found a weak dependence of medial gastrocnemius volume to body mass ratio on subject age in TD children and in a group of children with bilateral CP between 2 and 9 years of age [19]. However, the results of Barber et al. imply that age is a weak factor of the muscle volume to body mass ratio, but do not investigate the relationship between age, body mass, and muscle volume. Therefore, comparison of the current study with the results of Barber et al. is difficult due to the different analysis techniques employed and the limitation of investigating the relationship between muscle volume normalised to body mass with age when body mass and age are strongly correlated. It remains a possibility that age is indeed a significant independent factor for lower limb muscle growth in this period of childhood, and further investigations are merited. In contrast, the results of Herskind et al. are remarkably similar to our own in spite of the large difference of the age ranges of the subjects in the two studies. They found that gastrocnemius muscle volume was highly correlated with body mass (and also age, height and fibula length) in groups of infants with and without CP, and the slope of the relationship was significantly reduced in the group with CP [18]. Our results and those of Herskind suggest an altered trajectory of muscle growth in individuals with CP. This altered relationship between muscle size and body mass may, in part, explain the decrease in the ratio of voluntary muscle and body mass in children and adolescents with CP [9].

The implications of reduced muscle growth on an individual’s ability to perform a functional task can be understood more easily by investigating lower limb muscle volume normalised to body mass. The calculated lower limb muscle volume from the lines of best fit in Fig. 1 for a TD individual a CP individual, gives a total lower limb muscle volume normalised to body mass of 41.3 mlkg^−1^ and 35.9 mlkg^−1^ at 40 kg body mass respectively, with a difference of 5.4 mlkg^−1^ between the groups; and 51.4 mlkg^−1^ and 37.3 mlkg^−1^ at 80 kg body mass respectively, with a 14.1 mlkg^−1^ difference between the groups. Compared to the TD normalised muscle volume at each body mass, the CP normalised muscle volume is reduced by 13% at 40 kg, and by 27.4% at 80 kg. It is possible that this large relative reduction in normalised muscle volume between body masses of 40 and 80 kg may have a significant impact on an individual’s ability to perform activities of daily living. Muscles of the lower limb are required to perform many of the activities of daily living and deficits in volume have an impact on the forces and powers that they can develop. Many activities of daily living require significant muscular forces (for example, moving without support from a seated to a standing position). If deficits in muscle volume are large enough, individuals may not be able to perform these tasks or have an adequate muscular reserve to perform them repeatedly [21]. Typically developing adults experience a decline in skeletal muscle mass in later life [22, 23], with low skeletal muscle mass relative to body mass (sarcopenia) being related to reduced function [23]. Individuals with cerebral palsy typically experience a decline in function with age. In cohort studies, 22% of individuals aged 10 years [7], 25% aged 25 years [7], and 75% of individuals aged 60 years [8], experienced a decline of at least one level of mobility or had died within 15 years. Although decline in function in later life in CP appears to be associated with the level of neurological impairment and the timing of the acquisition of early motor milestones [24] these factors do not explain the biomechanical or pathophysiological causes of the loss of mobility typically experienced by individuals with CP. The increasing deficit in skeletal muscle size relative to body mass observed in this study may, in part, explain the decline in function with age. As individuals with reduced muscular reserve go through adulthood, the natural history of decline in muscle performance with age may contribute to their established muscle deficits, reducing further their muscular reserve, and compromising their mobility.

Although the results of this study aid in understanding muscle growth in this population, the clinical implications of these results are unclear. Previous studies in cohorts of children with CP show that muscle volume can be increased by resistance training, the role of strength training for altering the trajectory of the relationship between lower limb muscle volume and muscle mass in the long-term remains unclear. Longitudinal cohort studies are required to confirm the results of this cross-sectional study, to establish if reductions in muscle volume are related to deterioration in gross motor function, and to investigate whether muscle performance and gross motor function can be altered by clinical interventions, such as strength training or pharmaceutical interventions.

The deficit in muscle volume with increasing body mass in CP may also be dependent on GMFCS level. Lower limb muscle volumes have previously been shown to have greater deficits with increasing GMFCS level compared to their TD peers [12, 17]. Although, this study was not designed to capture differences in the relationship between muscle volume and body mass between groups of individuals at different GMFCS levels, we retrospectively investigated the effect of GMFCS level on muscle volume. Of the individuals with CP that participated in this study, six individuals were categorised GMFCS level I, fourteen GMFCS level II, and five GMFCS level III. The relationships between muscle volume and body mass for the individuals with CP stratified by GMFCS level are depicted in the Additional file 1. Regression analysis was not performed for the individuals with GMFCS III as four of the five data points were clustered. The slopes of the linear regression model were 5.4% (GMFCS I) and 46.9% (GMFCS II) shallower than the slope of the TD group. This result suggests that the trajectory of muscle growth in bilateral CP may be related to the severity of the motor disability.

### Study limitations

This study is cross-sectional in design and therefore the relationships observed in this study may not be representative of longitudinal muscle growth for individual subjects. This study may provide evidence to support future longitudinal muscle growth studies in this patient group.

We chose to test the relationship between muscle volume and body mass. However, Handsfield et al. made the case that the bodymass*height product may explain muscle volume more fully than body mass alone [11], however they did not show a significant difference between the two proposed explanatory variables. Similarly, in our study, the body mass-height product had no significant statistical advantage over body mass alone (see Additional file 1). Similar results were obtained when muscle volume is scaled with height-mass product instead of body mass. For completeness, the analysis of muscle volume with body mass and height-mass product is provided in the Additional file 1.

A constant (intercept) was included in the statistical analysis of the relationship between muscle volume and body mass between the groups. The constant in the regression equation for the TD group was −786.61 with 95% a confidence interval of −1761.31 to 188.09. The constant for the Group with CP was 26.90 with 95% a confidence interval of −525.59 to 471.79. Although the origin is within the confidence interval of the constant for both subject groups, this does not preclude there being a genuine non-zero intercept in the data. Repeating the analysis of our data with a zero constant (through the origin), a fixed ratio of muscle volume to body mass is obtained, 50.907*body mass for the TD group and 36.701*body mass for the Group with CP. This means that there would be a constant percentage difference in muscle volume normalised to body mass between the groups of 28%. However, it is common practice to include a constant in regression models, even when the constant is not statistically significant. The value of the constant is a prediction for muscle volume at zero body mass. Although this appears appropriate for muscle volume and body mass, data was not collected in this data range, and the relationship between muscle volume and body mass may be very different at very low body mass. Fixing the constant at zero may add a significant artefact to the data. The size and mass range of the individuals in the current study prevents the value of the intercept to be explored fully. Herskind et al. reported a non-zero constant for both the CP and TD groups for the relationship between medial gastrocnemius muscle volume and body mass in children aged 8 to 65 months [18], which supports the inclusion of a non-zero constant in the analysis performed in this study.

As participants in this study were aged between 10 and 23 years, it is possible that puberty and maturation may have influenced the results. However we observed that age was not a significant independent explanatory factor of muscle volume for the participants under 17 years of age (see Additional file 1). Age, sex, and body mass were included as covariates in our statistical analysis (ANCOVA). However, age and sex were not found to be significant independent explanatory factors of muscle volume. It is possible that pubertal stage may explain greater variance of muscle volume in adolescence compared to age. Any changes in muscle volume relative to body mass during puberty will add variation to the dataset depending on the variable pubertal stage of the volunteers in the study. Despite this strong linear regression coefficients were observed between lower limb muscle volume and body mass in both subject groups.

Growth hormone (GH) levels were not investigated in this study. It is possible that GH levels may explain some of the variability in muscle size observed in this study. Considering that GH deficiency is common in individuals with CP [25], GH level may be an explanatory factor for the reduced relationship between muscle size and body mass in CP observed in this study and this requires further investigation.

Muscle volume can vary with ethnicity. The majority of participants in this study were white caucasian (24 in each group). Although the ethnicities of the participants in this study were similar between groups enabling a comparison to be performed, caution should be used when extrapolating the results of this study to other ethnicity groups.

The majority of participants with CP in this study have previously undergone interventions as part of their routine clinical management. It is possible that the diverse histories of surgical intervention, serial casting, botulinum toxin injections and physiotherapy received by the individuals with CP in this study may have influenced the development of particular muscles. Therefore, the results of this study may not be completely representative of the natural history of muscle development in CP. However, it is not possible to obtain a fully representative group of young people with CP who have not received some significant intervention affecting their lower limbs.

MRI data was collected using three different MRI protocols on two scanners. The different protocols resulted in different images, particularly between the T1 weighted and Dixon sequences. However, all sequences produced good image contrast between muscles, and the muscle boundaries could be easily identified. Muscle volume measurements are geometric measurements that are standardised across MRI imaging systems.

## Conclusions

This cross-sectional study suggests that muscle growth is reduced relative to body mass in adolescents and young adults with CP. However, a longitudinal study is required to further investigate altered muscle growth trajectories in this group and their impact on long-term mobility.

## Additional file

Additional file 1: 1. The scaling of lower limb muscles volume to height and body mass. Description of data: A comparison of the power of height, body mass, and height-mass product to explain muscle volume. 2. The relationship between muscle volume and height-mass product. Description of data: A repeat of the analysis of covariance performed in the manuscript to investigate differences between the subject groups using height-mass product instead of body mass as a covariate of muscle volume. 3. The relationship between muscle volume and body mass for participants under 17 years of age. Description of data: A repeat of the analysis of covariance performed in the manuscript to investigate differences between the subject groups using body-mass as the covariate of muscle volume, but with only the subjects aged under 17 years included in the analysis. 4. The relationship of muscle volume to body mass stratified by GMFCS level, Participant intervention history. Description of data: To investigate whether the deficit in muscle volume with increasing body mass in CP may also be dependent on GMFCS level, linear regression and ANOVA were repeated for the CP group stratified by GMFCS level. 5. Participant intervention history. Description of data: A table of the intervention history for each participant. (DOCX 33 kb)

## Abbreviations

ANCOVA: Analysis of co-variance
BMI: Body Mass Index
CP: Cerebral palsy
GMFCS: Gross motor function classification system
ICC: Intra-class correlation coefficient
MDC: Minimal detectable change
MRI: Magnetic resonance imaging
SEM: Standard error of measurement
TD: Typically developing
