# Supplementary material for: The relationship between lower limb muscle volume and body mass in ambulant individuals with bilateral cerebral palsy
Source: BMC Neurol. 2017 Dec 29;17:223. doi: 10.1186/s12883-017-1005-0 (PMC5747099; doi:10.1186/s12883-017-1005-0)
Supplement: Additional file 1: — 1. The scaling of lower limb muscles volume to height and body mass. Description of data: A comparison of the power of height, body mass, and height-mass product to explain muscle volume. 2. The relationship between muscle volume and height-mass product. Description of data: A repeat of the analysis of covariance performed in the manuscript to investigate differences between the subject groups using height-mass product instead of body mass as a covariate of muscle volume. 3. The relationship between muscle volume and body mass for participants under 17 years of age. Description of data: A repeat of the analysis of covariance performed in the manuscript to investigate differences between the subject groups using body-mass as the covariate of muscle volume, but with only the subjects aged under 17 years included in the analysis. 4. The relationship of muscle volume to body mass stratified by GMFCS level, Participant intervention history. Description of data: To investigate whether the deficit in muscle volume with increasing body mass in CP may also be dependent on GMFCS level, linear regression and ANOVA were repeated for the CP group stratified by GMFCS level. 5. Participant intervention history. Description of data: A table of the intervention history for each participant. (DOCX 33 kb) [file 12883_2017_1005_MOESM1_ESM.docx]

**1. Scaling of lower limb muscles volume to height and body mass**

In the literature a case has been made that the product of height and body mass may be a more powerful explanatory variable of lower limb muscle volume than body mass alone. Below we compare the power of height, body mass, and height-mass product to explain muscle volume in our dataset.

Linear regression was performed to assess the linear relationship of limb muscle volume with body mass, height, and the height-mass product in the TD group. A two-tailed t-test of Fisher transformation to compare the effectiveness of height, body mass, and height-mass product scaling with lower limb muscle volume. Lower limb muscle volume in the TD group was significantly linearly related to height (R^2^=0.70, *p*<0.001), body mass (R^2^=0.77, *p*<0.001), and height-mass product (R^2^=0.80, *p*<0.001) a shown in figures S.1 to S.3. However, no significant difference was observed between any of linear regression coefficients (height-mass product vs. body mass: *z*=0.23, *p*=0.818; height-mass product vs. height: *z*=0.74, *p*=0.459; body mass vs. height: *z*=0.51, p=0.610).

|  |
| --- |
| **Figure S.1**: Lower limb muscle volume against height for the CP group (circle, dashed line, R^2^=0.70, muscle volume = 4313*height - 4966) and TD group (square, sold line R^2^=0.70, muscle volume = 5721*height -6568). |
|  |
| **Figure S.2**: Lower limb muscle volume against body mass for the CP group (circle, dashed line, R^2^=0.75, muscle volume = 37.2*body mass - 26.9) and TD group (square, sold line R^2^=0.77, muscle volume = 62.57*body mass - 786.6). |
|  |
| **Figure S.3**: Lower limb muscle volume against height-mass product for the CP group (circle, dashed line, R^2^=0.79, muscle volume = 19.4*height-mass product + 265.9) and TD group (square, sold line R^2^=0.80, muscle volume = 28.2* height-mass product + 88.0). |

**2. The relationship between muscle volume and height-mass product**

As noted above, in the literature a case has been made that the product of height and body mass may be a more powerful explanatory variable of lower limb muscle volume than body mass alone. Below we repeat the analysis of covariance (ANCOVA) analysis of muscle volume using height-mass product instead of body mass a covariate.

Figure S.3 shows lower limb muscle volume against height-mass product for the CP and TD groups. The slope of the linear regression equation for the CP group was 31.2% smaller compared to the TD group (see table S.1). Performing an ANCOVA found that lower limb muscle volume is significantly related to body mass (p<0.001) and that there is a significantly difference between the slopes of the linear relationships of the two subject groups (*p*=0.007), with subject group (*p*=0.238), sex (*p*=0.108), and age (*p*=0.406) found to not be significant factors of lower limb muscle volume.

**3. The relationship between muscle volume and body mass for participants under 17 years of age**

As participants in this study were aged between 10 and 23 years, it is possible that puberty and maturation may have influenced the results. Although age was observed to not be a significant independent explanatory factor of muscle volume, we repeated the analysis in a subset of the participants who were under 17 years of age, who had therefore not reached skeletal maturity.

Pearson’s correlations were utilised to test for linearity between body mass and muscle volumes and Analysis of co-variance (ANCOVA) was employed to investigate whether lower limb muscle volume was significantly related to subject group, age, or body mass in a subset of the participants who were under 17 years of age. The ANCOVA had subject group and sex as fixed factors and age and body mass as covariates with the model including the interaction between subject group and body mass to test whether there was a significant difference in the slopes of the relationship of lower limb muscle volume to body mass between the groups.

Figure S.4 shows lower limb muscle volume against body mass for the CP and TD groups under 17 years of age. Muscle volume and body mass were linearly related in the TD group (R^2^=0.86, p<0.001) and in the CP group (R^2^=0.64, p<0.001). Performing an ANCOVA found that lower limb muscle volume is significantly related to body mass (p<0.001) and that there is a significantly difference between the slopes of the linear relationships of the two subject groups (*p*=0.009), with subject group (*p*=0.198), sex (*p*=0.513), and age (*p*=0.098) found to not be significant factors of lower limb muscle volume.

|  |
| --- |
| **Figure S.4:** Lower limb muscle volume against body mass for a subset of subjects aged less than 17 years in the CP group (circle, dashed line, R^2^=0.64, muscle volume = 34.4*body-mass + 92.46) and TD group (square, sold line R^2^=0.86, muscle volume = 70.4* body-mass - 1137.1). |

**4. The relationship of muscle volume to body mass stratified by GMFCS level**

To investigate whether the deficit in muscle volume with increasing body mass in CP may also be dependent on GMFCS level, linear regression and ANOVA were repeated for the CP group stratified by GMFCS level. The CP group were a convenience sample of individuals attending clinics at our university hospital, which resulted in an unequal number of participants at each GMFCS level, with six individuals who are GMFCS level I, fourteen GMFCS level II, and five GMFCS level III. Significant linear relationships were observed for the GMFCS I (R^2^=0.81, *p*<0.015) and II (R^2^=0.86, P<0.001) (Figure S.5). Linear regression was not performed for the GMFCS III group as 4 out of the 5 subjects at GMFCS III were clustered together, as can be seen in Figure S.5. The slopes of the slope of the linear regression model were 5.4% and 46.9% smaller than the gradient of the TD group for the GMFCS I and II subgroups respectively. Performing an ANOVA found that the difference in the between the slopes of the GMFCS I subgroup was not significantly different from the TD group (*p*=0.902) but there was a significant difference between the GMFCS I and II (*p*=0.044) and between the GMFCS II and TD groups (*p*=0.002).

|  |
| --- |
| **Figure S.5:** Lower limb muscle volume against body mass for the TD group (cross, grey starred line, R^2^=0.77, muscle volume = 62.57*body mass -786.6) and CP group stratified by GMFCS level: GMFCS level I (diamond, solid line, R^2^=0.81, muscle volume = 59.8*body mass - 904.5); GMFCS level II (square, dashed line, R^2^=0.86, muscle volume = 33.2*body mass + 176.6) and GMFCS level III (triangle, dotted line, R^2^=0.86, muscle volume = 37.2*body mass + 176.6). |

**5. Participant intervention history**

| Subject | Surgery | Botulinum toxin injection |
| --- | --- | --- |
| 1 | Bilateral Achilles tendon and hamstring lengthening. Repeat right hamstring lengthening | None |
| 2 | None | None |
| 3 | Bilateral psoas and hamstring lengthening. Repeat Bilateral medial hamstring lengthening and bilateral rectus femoris transfer | Bilateral gastrocnemius (x2). Bilateral hamstrings |
| 4 | Right femoral derotation osteotomy. Bilateral hamstring and calf lengthening. | None |
| 5 | Left hamstring lengthening | Left gastrocnemius (x2) |
| 6 | None | None |
| 7 | None | None |
| 8 | Right tibialis posterior, Achilles tendon, FDL & FHL lengthening | Right gastrocnemius |
| 9 | Bilateral hamstring and gracilis lengthening. Right calf lengthening, Achilles tendon lengthening. | Right gastrocnemius and hamstrings |
| 10 | Bilateral hamstring lengthening. Left ST tendon transfer to SM. Left BFLH and Achilles tendon lengthening. | Bilateral gastrocnemius (x4) |
| 11 | Left calf, gracilis, and hamstring lengthening | None |
| 12 | Right femoral derotation osteotomy and right calf lengthening | None |
| 13 | None | None |
| 14 | None | None |
| 15 | Bilateral lateral column lengthening and calf lengthening. Left hamstring lengthening. Bilateral Adductor release. Repeat bilateral hamstring lengthening | Bilateral hip adductors |
| 16 | Bilateral hamstring and calf muscle lengthening and bilateral calcaneal osteotomy | Bilateral hip adductors |
| 17 | Right calf lengthening | None. |
| 18 | Bilateral hip adductor release | Hip adductors (x3) |
| 19 | Bilateral hip adductor release | None. |
| 20 | Bilateral hamstring and calf lengthening | None. |
| 21 | Bilateral calf lengthening | None. |
| 22 | Bilateral lateral column lengthening and calf lengthening with left hamstring lengthening | None |
| 23 | Bilateral hamstring and calf muscle lengthening and bilateral calcaneal osteotomies | None. |
| 24 | Right cavo-varus foot deformity correction | None |
| 25 | Bilateral hamstring and calf lengthening | None |
| Table S.1: Intervention history for all participants in the CP group. (x2,x3,x4) denotes number of botulinum toxin injections. | | |
